# Integrative Role of Yoga and Naturopathy in Cancer Rehabilitation: A Narrative Review

**DOI:** 10.7759/cureus.105544

**Published:** 2026-03-20

**Authors:** Vadde Venkata Karthik, Hema Chaitanya Latha Reddy, Harini Rudramurthy, Karthik P Hongal, Indira Lakshmi Putta, Charulatha KTK, Shivaprasad Shetty, Sujatha KJ

**Affiliations:** 1 Department of Yoga Therapeutics, Shri Dharmasthala Manjunatheshwara (SDM) College of Naturopathy and Yogic Sciences, Ujire, IND; 2 Department of Natural Therapeutics, Shri Dharmasthala Manjunatheshwara (SDM) College of Naturopathy and Yogic Sciences, Ujire, IND

**Keywords:** cancer rehabilitation, cancer-related fatigue, naturopathy, quality of life, yoga therapy

## Abstract

Cancer poses a significant global health challenge, with survivors often dealing with ongoing fatigue, pain, sleep issues, and psychological distress despite advances in traditional treatments. Integrative oncology has increasingly included yoga and naturopathy to address these complex needs and enhance quality of life. We conducted a narrative review of English-language studies indexed in PubMed and Scopus between 2000 and 2025, using the keywords “yoga,” “cancer-related fatigue,” “cancer,” “cancer-related quality of life,” “cytokines,” “naturopathy,” and “cancer rehabilitation.” We included randomized controlled trials, clinical trials, and reports on mechanistic or integrative programs in adult oncology populations; non-peer-reviewed, non-indexed sources were excluded. Yoga interventions, generally a mix of asanas, pranayama, and meditation, have been shown to reduce cancer-related fatigue and improve mood, sleep, and overall quality of life, especially in breast and mixed-cancer groups, with small-to-moderate effects noted in meta-analyses. Mechanistic studies reveal reductions in cortisol and pro-inflammatory cytokines such as interleukin (IL)-1β and IL-1ra, indicating modulation of chronic inflammation and stress responses. Naturopathy-based programs, combining diet, hydrotherapy, massage, and lifestyle counseling, offer additional benefits in functional capacity, symptom relief, and patient-reported outcomes alongside conventional treatments. Overall, yoga and naturopathy are generally safe when properly adapted, with increasing feasibility for both group and home-based formats. Evidence supports yoga and emerging naturopathy-inclusive programs as safe, effective adjuncts for cancer rehabilitation, especially for fatigue, distress, sleep issues, and inflammation. Larger, mechanistically focused trials on combined yoga-naturopathy approaches are needed to improve personalization and implementation in routine cancer care.

## Introduction and background

Cancer is a major global health challenge, with an estimated 19.3 million new cases and almost 10 million deaths reported worldwide in 2020 [1,2]. Beyond survival, cancer and its treatments impose substantial physical (pain, fatigue, nausea, immunosuppression), psychological (anxiety, depression, cognitive difficulties), and social (isolation, economic stress, role disruption) burdens that markedly impair quality of life across the disease trajectory [3-5]. Although modern treatments such as surgery, chemotherapy, radiotherapy, and targeted therapies have markedly improved survival, delayed disease progression, and reduced cancer-related deaths, many people still live with ongoing health problems long after treatment ends. Survivors commonly report persistent cancer-related fatigue, chronic pain, sleep difficulties, nerve-related symptoms, problems with memory and concentration, low mood, anxiety, sexual and emotional concerns, and an overall decline in day-to-day quality of life [6]. This has driven growing interest in complementary and integrative medicine approaches that aim to enhance conventional care by improving symptom control, psychological resilience, and overall well-being [7]. Among these, yoga is one of the most widely accepted and studied modalities in integrative oncology, and, together with naturopathy, offers a promising holistic strategy to address multidimensional needs and improve quality of life in cancer rehabilitation [8]. Yoga, involving asanas, pranayama, and meditation, reduces cancer-related fatigue, anxiety, and inflammation via neuroendocrine modulation. Naturopathy, emphasizing hydrotherapy, mud therapy, nutrition, and massage, complements this by targeting detoxification, pain relief, and immune support, as seen in integrative protocols for advanced cancers [9].

The objective of this review is to assess the role of yoga and naturopathy in cancer rehabilitation, with an emphasis on symptom management, quality of life, and mechanistic pathways. Specific goals are to (i) summarize clinical outcomes associated with yoga in cancer populations, (ii) outline the contribution of naturopathic interventions in oncologic settings, (iii) explore mechanistic intersections along neuro‑immune‑metabolic axes, and (iv) discuss implications for practice and research. By examining these domains collectively, the review aims to clarify how an integrated yoga-naturopathy framework can support a more patient‑centric model of cancer rehabilitation.

## Review

Methodology

A focused literature search was conducted in PubMed and Scopus for studies published between 2000 and 2025. The search was restricted to English-language, full-text publications involving adult human participants with a current or prior cancer diagnosis. We combined relevant keywords and Medical Subject Headings (MeSH) using Boolean operators, for example, (“yoga” OR “mind-body interventions”) AND (“cancer-related fatigue” OR “fatigue”) AND (“cancer” OR “oncology”) AND (“quality of life” OR “cancer-related quality of life”) AND (“cytokines” OR “inflammation” OR “immune markers”) AND (“naturopathy” OR “integrative oncology” OR “cancer rehabilitation”). Eligible studies included randomized controlled trials (RCTs), controlled clinical trials, mechanistic studies assessing inflammatory or immune biomarkers, and integrative clinical program reports that evaluated yoga- or naturopathy-based interventions in adult oncology populations and reported outcomes related to cancer-related fatigue or quality of life. We excluded pediatric and animal studies, non-peer-reviewed sources, conference abstracts, non-English-language publications, and studies that did not report fatigue or quality of life outcomes. Titles and abstracts were screened for relevance, followed by full-text review against predefined inclusion and exclusion criteria. The initial search yielded 164 records (146 from electronic databases and 18 from manual searches). After removal of 32 duplicates, 132 unique records were screened by title and abstract, of which 97 were excluded due to lack of relevance, non-interventional design, animal or pediatric samples, or absence of cancer-related fatigue or quality of life outcomes. A total of 35 full-text articles were assessed in detail, and 22 were excluded because they did not report the outcomes of interest. A total of 13 studies met all inclusion criteria and were retained for this narrative review, comprising RCTs, clinical intervention studies, qualitative research, and integrative oncology program reports evaluating yoga- and naturopathy-based interventions in adult patients with cancer.

Yoga‑based interventions in cancer rehabilitation

Cancer-Related Fatigue

Approximately 80% of individuals suffer from moderate-to-severe cancer-related fatigue either while receiving treatment or following completion of treatment. As such, cancer-related fatigue represents one of the most commonly reported debilitating symptoms experienced by individuals with cancer [3]. Cancer-related fatigue interferes with daily activities, treatment compliance, and quality of life, but is difficult to manage effectively using conventional approaches alone. Yoga has been identified as a powerful non-pharmacologic tool for managing cancer-related fatigue through its influence on physical conditioning, autonomic function, and psychosocial support [10]. Several RCTs and meta-analyses support the effectiveness of yoga in reducing cancer-related fatigue. A systematic review including 13 RCTs showed that yoga resulted in moderate reductions in fatigue in breast cancer survivors (standardized mean difference (SMD) = -0.54). Follow-up studies have since supported these results in patients with a variety of cancers, including breast, colorectal, lung, prostate, and hematologic cancers, showing that yoga reduces fatigue scores significantly compared with usual care or health education controls. These effects persist beyond the intervention period, highlighting sustained benefit [11].

Pain and Physical Function

Cancer pain, often complex and undertreated, results from tumor invasion, toxic effects of cancer therapies, and associated diseases [12]. Yoga’s gentle range of motion postures and mindful breathing enhance musculoskeletal flexibility, improve venous and lymphatic return, and activate parasympathetic pathways, which together may reduce central sensitization, downregulate stress-responsive neuroendocrine circuits (such as the hypothalamic-pituitary-adrenal axis), modulate inflammatory cytokines, and increase endogenous pain-inhibitory mechanisms, thereby decreasing perceived pain and disability [13]. Clinical trials using standardized patient-reported pain measures have shown substantial reductions in chronic pain among cancer survivors practicing yoga. Notably, yoga practice is also helpful in improving functional status and decreasing stiffness, enabling greater autonomy in self-care [14].

Sleep Disturbances

Sleep disturbances are highly prevalent in oncology, and commonly include difficulty falling asleep, frequent night-time awakenings, early-morning awakening, non-restorative sleep, circadian rhythm disruption, and, in some cases, restless legs, all of which can worsen fatigue, mood, and immune function [15]. Yoga combines breathing techniques, gentle physical postures, and meditative practices that reduce hyperarousal of the sympathetic nervous system, enhance parasympathetic tone, stabilize circadian rhythms, and lower stress-related inflammatory signaling, thereby shortening sleep-onset latency, decreasing night-time awakenings, improving sleep efficiency, and promoting deeper, more restorative sleep. RCTs have shown effectiveness in reducing sleep-onset latency and overall sleep disturbances in patients with breast and prostate cancer [16]. These benefits are commonly associated with a reduction in symptoms of anxiety and depression, promoting a positive feedback loop that enhances overall well-being [17].

Psychological and Emotional Well-being

Cancer diagnosis and management also carry significant psychological tolls in terms of depression, anxiety, distress, and fear of recurrence [18]. Yoga’s meditative and mindfulness-based approach to emotional regulation promotes acceptance and resilience. Systematic reviews have found significant reductions in anxiety (SMD = -0.61) and depression (SMD = -0.48) in cancer patients undergoing yoga therapy compared to controls [19]. Group yoga therapy also offers social support, reduces feelings of isolation, and improves spiritual well-being [20].

Quality of Life

In addition to personal symptomatic management, yoga practice has been shown to enhance various aspects of health-related quality of life, such as physical, emotional, social, and role functioning [21]. Meta-analytic results show that yoga has a moderate positive effect on global quality of life, with more pronounced effects noted in patients undergoing longer duration interventions (≥8 weeks). These positive effects can be directly attributed to improved treatment tolerance, enhanced physical activity, and greater patient empowerment, important predictors of long-term survivorship [22].

Breast cancer survivors have been the main subject of interest for most yoga studies in the cancer population because of their relatively high incidence and interest in complementary approaches [23]. However, an increasing body of evidence now supports the safety and efficacy of yoga in other cancers such as prostate, colorectal, lung, blood, and pediatric cancers [24]. Multi-center clinical trials conducted in geographically, culturally, and infrastructurally diverse settings have established the adaptability and applicability of yoga [25].

Clinical Personalized Intervention Protocols

Yoga therapy in oncology is very flexible, with methods being particularly tailored to suit each patient’s individual medical condition, cancer type, stage, treatment phase, and prevailing symptoms. Such personalized interventions can be highly variable in several ways, including duration, intensity, frequency, and method of delivery. For patients with limited mobility or advanced disease, chair yoga or restorative yoga is a safe and effective starting point, using modified poses that avoid exacerbating symptoms while still promoting circulation and relaxation [26]. For cancer survivors who have regained their functional capacity, moderate-intensity yoga styles such as Hatha or Iyengar yoga are commonly practiced, which combine physical conditioning with breathing and mindfulness techniques to manage fatigue, musculoskeletal debility, and psychological distress [27]. Contemporary oncology yoga practice has also been extended to include home and tele-yoga programs to improve accessibility for patients with geographical, mobility, or immunocompetence limitations. Such digital and hybrid platforms enable regular patient engagement, facilitate mass dissemination, and enable personalization through interactive guidance and progress tracking, thus obviating the need for transportation and immunosuppression-related limitations [28]. Careful individualized assessments by cancer-specialized yoga therapists also ensure safety, appropriate levels of intensity, and smooth integration with simultaneous medical therapies, making yoga a holistic support modality throughout the cancer experience (Table 1).

**Table 1:** Tailoring yoga interventions in oncology. This table summarizes how yoga protocols in oncology are commonly adapted to different clinical situations, matching the style and intensity of practice to patient mobility, treatment phase, and overall functional status.

Situation/Patient type	Yoga approach	Main purpose
Advanced disease/Low mobility	Chair or restorative yoga	Gentle movement, circulation, relaxation, pain, and stiffness relief
During or soon after treatment	Gentle breathing practices	Symptom relief (fatigue, sleep, mood) and basic strength
Functionally recovered survivors	Hatha or Iyengar-based yoga	Fitness, flexibility, fatigue and stress management, and quality of life
Home or tele-yoga users	Online/Hybrid sessions	Access, regular practice, and safe guidance from home

Naturopathy in oncologic rehabilitation

Naturopathic medicine practices a broad spectrum of modalities, but some have been investigated in peer-reviewed oncology literature. A prospective cohort study on naturopathic oncology practice in thoracic malignancies outlined individualized treatment programs incorporating dietary counseling, exercise, stress management, acupuncture, and supplementation, showing high patient satisfaction and suggestive benefit for symptom control, although survival benefit was not established [29]. In another study on prostate cancer patients undergoing radiotherapy, additional naturopathic and nutritional supplements incorporating vitamins, minerals, and botanicals seemed to affect tumor response and quality of life, suggesting possible synergistic effects with conventional therapies [30].

Dietary-focused naturopathic therapies, usually incorporating plant-based, low-glycemic, and anti-inflammatory foods, aim to modulate metabolic factors driving malignancy and treatment toxicity [31]. By emphasizing vegetables, fruits, whole grains, legumes, nuts, and seeds, these approaches help reduce excess adiposity, improve lean mass-to-fat mass ratios, stabilize blood glucose and insulin responses, and lower systemic inflammatory markers, all of which are linked with better body composition, reduced fatigue, and more favorable metabolic profiles in cancer populations [32]. At a biochemical level, healthy dietary patterns can influence cytokine signaling, tending to lower pro-inflammatory mediators such as C-reactive protein, interleukin (IL)-6, and tumor necrosis factor-alpha (TNF-α), while supporting a more regulated immune environment that may enhance treatment tolerance and recovery. Micronutrients commonly emphasized in such protocols include antioxidant vitamins (A, C, E), vitamin D, B-complex vitamins, and trace elements such as zinc, selenium, and copper, as well as omega-3 fatty acids and arginine, which together support immune cell function, redox balance, and tissue repair. Diet also exerts profound effects on the gut microbiome. High-fiber, plant-forward diets increase beneficial taxa such as *Akkermansia*, *Faecalibacterium*, and other short-chain fatty acid-producing bacteria, whose metabolites (e.g., butyrate, propionate) nurture the intestinal barrier, modulate systemic immunity, and may improve responses to therapies including immunotherapy [33]. Mind-body and physical therapies used in naturopathic medicine, such as hydrotherapy (contrast baths, affusions), massage, therapeutic exercise, breathing practices, guided relaxation, and meditation, mirror rehabilitation goals by targeting pain modulation, circulatory enhancement, autonomic balance, and stress reduction, and are frequently combined with yoga in integrative oncology settings. Within relaxation therapy, naturopathic practice may incorporate techniques such as progressive muscle relaxation, guided imagery, mindfulness or mantra meditation, breathing-based relaxation, and sometimes music or aromatherapy, all aiming to downregulate sympathetic arousal and support parasympathetic recovery [34]. Conceptually, these naturopathic modalities and yoga share a common biopsychosocial framework, both seek to optimize internal milieu (inflammation, autonomic tone, endocrine balance), improve functional capacity, and enhance quality of life, and emerging scientific literature increasingly supports their complementary roles in comprehensive cancer rehabilitation. Evidence with more direct implications for the role of naturopathy in hard oncologic outcomes is beginning to appear in the literature from cohort and comparative trials. In a major observational study of patients with colon cancer treated with Chinese herbal compounds and vitamins in addition to chemotherapy, survival benefits were observed compared with standard care alone, supporting the hypothesis that well-designed herbal and nutritional approaches may influence treatment outcomes [35]. However, variability in preparation and the risk of herb-drug interactions emphasize the importance of standardized approaches.

Integrated yoga-naturopathy protocols

The integration of yoga and naturopathy in rehabilitation packages can be understood by the recent trials and cases. An RCT was conducted on patients with stage II and III adenocarcinoma of the colon. The adjuvant treatment program included yoga classes, naturopathic diet counseling, and certain physical nature-cure practices along with chemotherapy. The patients undergoing the integrative treatment showed improvement in hemoglobin levels; reduction in anxiety and depression; reduction in symptoms, including pain, fatigue, nausea, appetite loss, sleep disturbance, and treatment-related discomfort; and higher scores on the Functional Living Index-Cancer compared to those undergoing chemotherapy alone. This shows not only symptomatic relief but also hematological improvement, suggesting that these programs can enhance the tolerability and efficacy of cytotoxic therapy [36].

A detailed case report on a patient with advanced rectal carcinoma showed the combination of yoga practices, including asanas, pranayama, relaxation, and meditation, with naturopathic therapies, such as mud packs, hydrotherapy, massage, and a plant-based diet during palliative chemotherapy. During the course of treatment, the patient demonstrated a significant reduction in pain, as measured by visual analog scales, and significant improvement in disease-specific quality of life parameters. The case report highlights the importance of integrative care in managing not only pain and symptoms but also emotional and existential issues, thereby providing a more dignified and comfortable experience of survivorship or end-of-life care [13].

Discussion

Integration of current evidence suggests that combining yoga and naturopathy offers a more person-centered model of cancer rehabilitation, shifting the focus from tumor control alone to long-term symptom relief, functional recovery, and quality of life. Standard oncologic treatments remain central for disease control, but they are not designed to fully address the chronic fatigue, pain, sleep disturbance, emotional distress, and physical deconditioning that many survivors continue to experience years after treatment. Yoga addresses this gap through practices involving breath, movement, and meditation; reduces perceived stress and autonomic hyperarousal, normalizes the hypothalamic-pituitary-adrenal axis activity; and improves parasympathetic (vagal) tone, which, in turn, modifies neuroendocrine and immune signaling relevant to both symptoms and disease biology. Mechanistic studies have shown that yoga can increase heart rate variability, lower resting cortisol, and reduce pro‑inflammatory cytokines such as IL‑6, TNF‑α, IL-1β, IL-1ra, and CRP, changes that are associated with reduced fatigue, better mood, and potentially improved immune surveillance [37,38]. Naturopathic interventions influence the same neuro‑immune‑metabolic axis. Diets rich in plant-based, low‑glycemic, and anti‑inflammatory foods help stabilize glucose and insulin dynamics, reduce visceral adiposity, and downregulate inflammatory signalling, which can ease fatigue and support healthier body composition and metabolic profiles. Many dietary protocols emphasise phyto­chemicals such as polyphenols, carotenoids, organosulfur compounds, and isothiocyanates, which modulate oxidative stress, Nrf2 and NF‑κB pathways, and tumor cell proliferation or apoptosis in preclinical models [39]. At the same time, attention to micronutrients (e.g., vitamins D, C, B‑complex, zinc, selenium, omega‑3 fatty acids) and gut‑supportive, fiber‑rich foods can improve immune competence and reshape the gut microbiome, enhancing production of short‑chain fatty acids that influence both systemic inflammation and brain function. Within naturopathy, mind-body and physical modalities such as hydrotherapy, massage, breathing and relaxation techniques, and other gentle physical therapies are conceptually aligned with rehabilitation goals of pain control, circulatory support, and stress reduction, and are often delivered alongside yoga in integrative oncology programs [40]. The immune-modulating effects of yoga’s parasympathetic stimulation, heart rate variability, and reduction of cortisol and inflammatory mediators may help improve immune function and resilience to treatments and fear of recurrence [41]. Naturopathic approaches that help regulate blood sugar, suppress overactive oxidative stress, and provide adequate micronutrient levels further support mitochondrial and tissue repair, possibly counteracting some of the metabolic disturbances that occur with cancer and its treatments [42]. When combined comprehensively, these approaches seem to provide a broader range of the “hallmarks” of survivorship challenges, i.e., chronic inflammation, immune suppression, psychological distress, and functional impairment, than any single approach used in isolation.

From a practical standpoint, existing studies show that yoga and naturopathy can be combined and delivered across multiple settings, including outpatient oncology clinics, inpatient rehabilitation units, and community-based survivorship programs. These services have been offered through group classes, one-to-one sessions, structured home-practice materials, and tele-yoga formats, which help overcome barriers related to distance, mobility limitations, and treatment schedules [43]. When provided by trained practitioners who adapt postures, intensity, and techniques to disease stage, comorbidities, and treatment-related vulnerabilities, reported safety profiles are generally favorable, with serious adverse events uncommon. Practical safeguards include modifying or avoiding certain practices in the context of bone metastases, severe anemia, neuropathy, cardiopulmonary compromise, or thromboembolic risk, and coordinating with the oncology team regarding potential herb-drug interactions or hemodynamic effects of more intensive naturopathic procedures [44].

Scaling up integrative yoga-naturopathy programs in cancer care holds considerable promise, but several practical challenges remain. Successful implementation would require trained professionals with expertise in oncology-focused yoga therapy and naturopathy, along with adequate infrastructure, time allocation within clinical settings, sustainable funding, and support from insurance systems. Additionally, clear referral pathways and interdisciplinary coordination are essential, particularly in busy oncology clinics where patient load and resource constraints may limit the integration of complementary care services. Patients face their own hurdles, such as travel hassles, costs, symptoms, work-family juggles, and cultural skepticism, especially in rural or low-resource regions. Clinicians hesitate without standardized protocols, herb-drug safety data, and proof of long-term wins and cost savings, calling for better education and cross-team collaboration.

Research gaps persist, with programs lacking robust trials and yoga studies suffering from small samples, varied methods, and short follow-ups that obscure optimal dosing and comparisons. Naturopathy often bundles too many elements for clear takeaways, and much work caters to motivated elites in elite centers, not everyday, diverse patients. Yet, mounting evidence from RCTs, meta-analyses, and clinic realities backs their role in easing fatigue, pain, sleep issues, mood, and quality of life via neuroendocrine, immune, and metabolic pathways. Large-scale studies are needed to tackle workforce, access, and safety to deliver tailored survivorship care and lighten the long-term toll of the treatment.

## Conclusions

The integration of yoga and naturopathy as organized adjuncts to conventional oncology represents a pragmatic, evidence-informed approach to comprehensive rehabilitation, concurrently addressing psychological, immune, and metabolic dysfunctions. With proper training and precautions, these modalities can significantly improve the quality of life and symptom management in cancer survivors.
